# Laparoscopic and endoscopic cooperative surgery for advanced gastric cancer as palliative surgery in elderly patients: a case report

**DOI:** 10.1186/s40792-021-01325-1

**Published:** 2021-11-15

**Authors:** Marie Washio, Naoki Hiki, Kei Hosoda, Masahiro Niihara, Motohiro Chuman, Mikiko Sakuraya, Takuya Wada, Hiroki Harada, Takeo Sato, Kiyoshi Tanaka, Takeshi Naitoh, Yusuke Kumamoto, Takafumi Sangai, Satoshi Tanabe, Keishi Yamashita

**Affiliations:** 1 Department of Upper Gastrointestinal Surgery, Kitasato University School of Medicine, 1-15-1 Kitasato, Minami-ku, Sagamihara, Kanagawa 252-0375 Japan; 2 Department of Gastroenterology, Kitasato University School of Medicine, 1-15-1 Kitasato, Minami-ku, Sagamihara, Kanagawa 252-0375 Japan; 3 Department of Lower Gastrointestinal Surgery, Kitasato University School of Medicine, 1-15-1 Kitasato, Minami-ku, Sagamihara, Kanagawa 252-0375 Japan; 4 Department of General-Pediatric-Hepatobiliary Pancreatic Surgery, Kitasato University School of Medicine, 1-15-1 Kitasato, Minami-ku, Sagamihara, Kanagawa 252-0375 Japan; 5 Department of Breast and Thyroid Surgery, Kitasato University School of Medicine, 1-15-1 Kitasato, Minami-ku, Sagamihara, Kanagawa 252-0375 Japan; 6 Division of Pediatric Surgery, Kitasato University School of Medicine, 1-15-1 Kitasato, Minami-ku, Sagamihara, Kanagawa 252-0375 Japan; 7 Division of Advanced Surgical Oncology, Department of Research and Development Center for New Medical Frontiers, Kitasato University School of Medicine, 1-15-1 Kitasato, Minami-ku, Sagamihara, Kanagawa 252-0375 Japan

**Keywords:** Elderly, Gastric cancer, Laparoscopic and endoscopic cooperative surgery, Palliative surgery

## Abstract

**Background:**

The number of elderly patients with gastric cancer is increasing, with the very elderly often refusing radical gastrectomy with lymph node dissection. Such a patient presented to us and we proposed a palliative surgery involving gastric local resection using laparoscopy endoscopy cooperative surgery (LECS).

**Case presentation:**

An 89-year-old woman presented to our hospital with progressing anemia. She had an aortic arch replacement for aortic dissection 6 months previously and was taking antithrombotic drugs for atrial fibrillation. She was diagnosed with advanced gastric cancer, and we presented a radical resection treatment plan involving distal gastrectomy with lymph node dissection. However, she strongly refused undergoing radical gastric cancer resection. We believed that at least local control of the tumor could be effective in preventing future bleeding or stenosis due to tumor progression. Therefore, we proposed a local gastrectomy with LECS as an optional treatment, and she agreed to this treatment. The surgery was performed with minimal blood loss, and no postoperative complications were observed. Histopathological examination revealed a 45 × 31-mm, Type 2, poorly differentiated adenocarcinoma (pT4a, ly0, v1a), and the resected margin was negative. The patient was alive 2 years after surgery without apparent recurrence or other illness. In addition, her weight was maintained, together with her daily activity.

**Conclusion:**

Local resection of gastric cancer with LECS might be an option for the palliative treatment of patients who refuse radical resection of gastric cancer.

## Background

Although gastric cancer treatment guidelines are useful for treatment selection in patients with gastric cancer, sometimes they are not applicable to those with surgical risk factors or older patients who do not have a long life expectancy [1]. Unfortunately, there are no current alternative therapies that bridge the gap between routine and palliative care. Laparoscopic and endoscopic cooperative surgery (LECS) is a procedure combining laparoscopic gastric resection with endoscopic submucosal dissection for local resection of gastric tumors. In 2006, Hiki et al. reported “classical LECS” and established it as a minimally invasive surgery for submucosal tumors [2]. Recently the procedure was also covered for gastric cancer by the Japanese National Health Insurance scheme. Partial gastrectomy with LECS results in minimal postoperative gastrectomy syndrome and minimizes postoperative physical weakness. Here, we report LECS as a palliative treatment for advanced gastric cancer (AGC) in an elderly patient who refused radical surgery.

## Case presentation

**Fig. 1:**
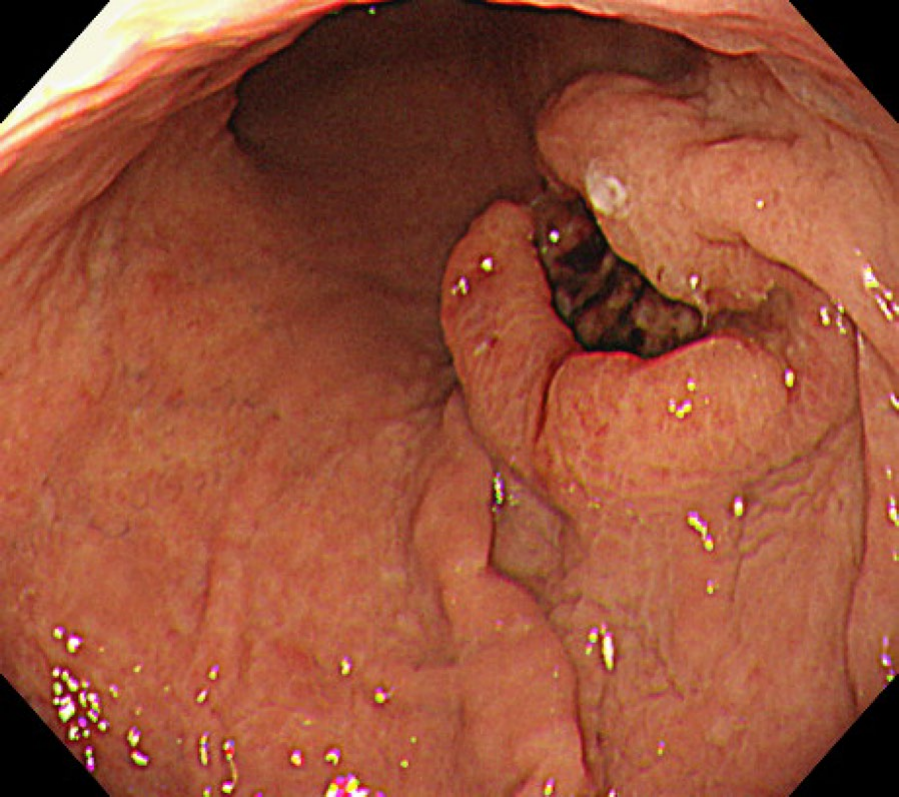
Gastroscopy revealed a Type 2 advanced gastric cancer at the posterior wall of the stomach

An 89-year-old woman presented to the hospital with progressing anemia. Gastroscopy revealed a Type 2 AGC at the posterior wall of the stomach (Fig. 1), which was diagnosed histologically as a poorly differentiated adenocarcinoma. Computed tomography showed no lymph node swelling or distant metastases. In summary, her clinical diagnosis was AGC, L, Post, 40 mm, Type 2, por., cT4aN0M0, cStage IIB (UICC 8th Edition).

As for her general condition, her activities of daily living were good, her Eastern Cooperative Oncology Group performance status was 0, and her Charlson comorbidity index was 7. Risk factors for surgery included a total aortic arch replacement for aortic dissection (Stanford A) 6 months previously, and comorbidities of atrial fibrillation, chronic heart failure (New York Heart Association functional classification II), and chronic kidney disease.

The standard treatment for this tumor would be distal gastrectomy with lymphadenectomy. However, the patient refused this option. Considering her high age, the option of LECS was suggested as palliative surgery, which she accepted.

**Fig. 2:**
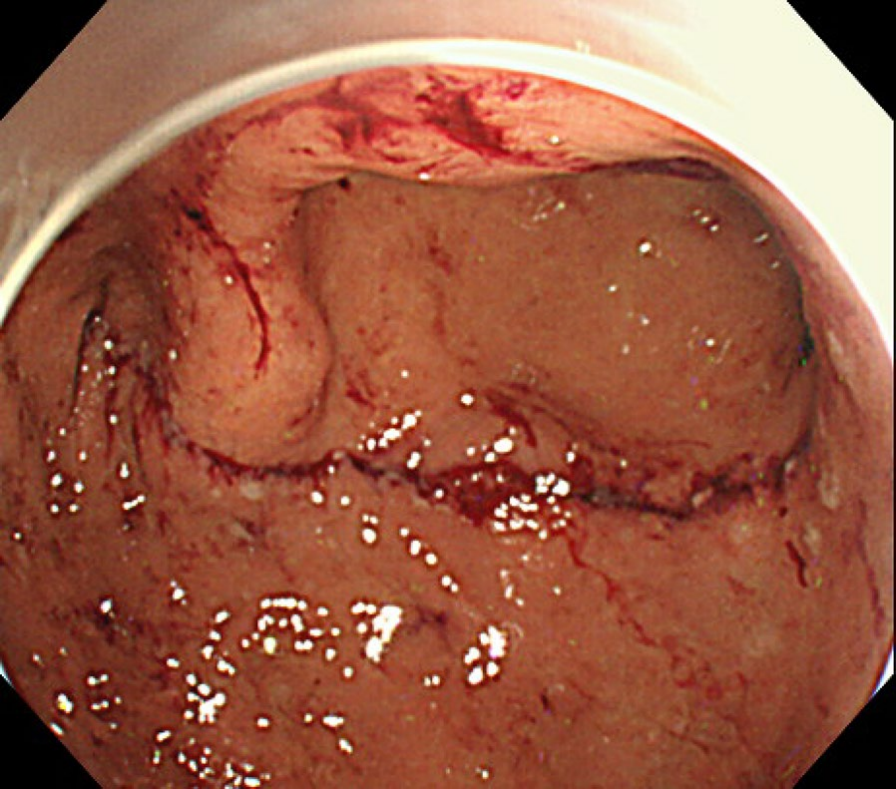
Intraoperative endoscopy for confirmation showed almost no deformation of the stomach

**Fig. 3:**
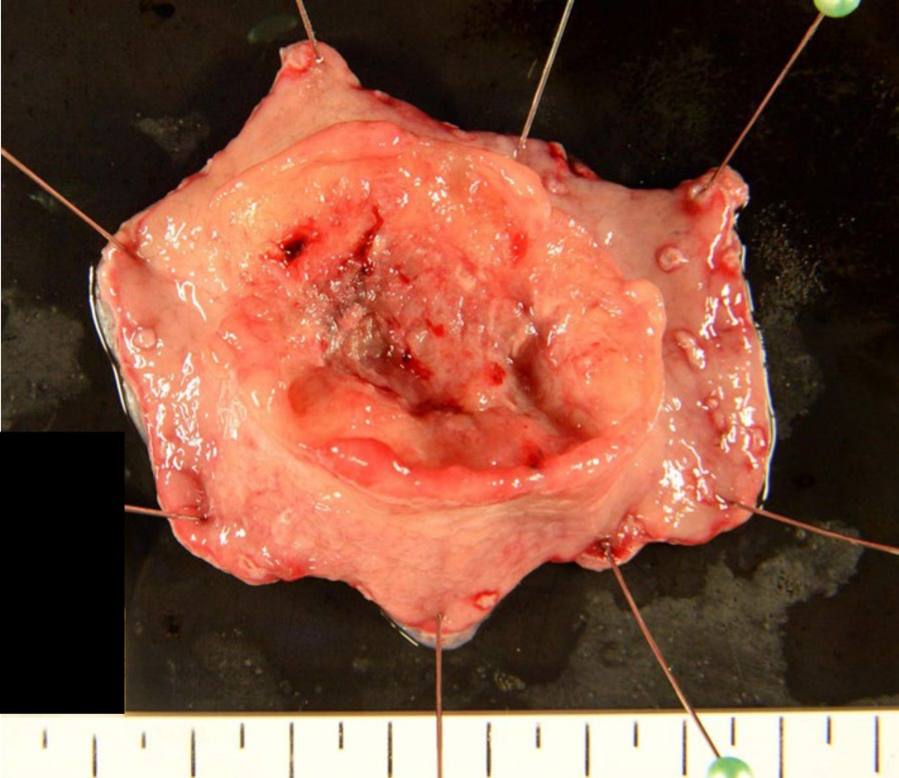
Postoperative pathologic examination demonstrated the following: 45 × 31-mm, Type 2, poorly differentiated adenocarcinoma (pT4a, ly0, v1a), with no lymph node metastases (0/6)

We performed an inverted-LECS using the crown method. The first intraperitoneal observation revealed no ascites, peritoneal dissemination, or distant metastases. Intraperitoneal lavage cytology was negative. An enlarged lymph node (#4d) was detected but the pathologic diagnosis was negative for malignancy. After endoscopic submucosal dissection, we placed sutures around the edges of the stomach incision and lifted the stomach wall ventrally like a crown shape. The tumor was resected endoscopically and dropped into the gastric cavity. After placing the tumor in a bag, the edge of the incision line was properly closed using a laparoscopic stapling device. Finally, we confirmed that there was no deformation of the stomach using endoscopy (Fig. 2). The operative time was 228 min, and the estimated intraoperative blood loss was 0 ml. The postoperative macroscopic and pathological diagnosis was a 45 × 31-mm, Type 2, poorly differentiated adenocarcinoma (pT4a, ly0, v1a), with no lymph node metastases (0/6) (Fig. 3).

**Fig. 4:**
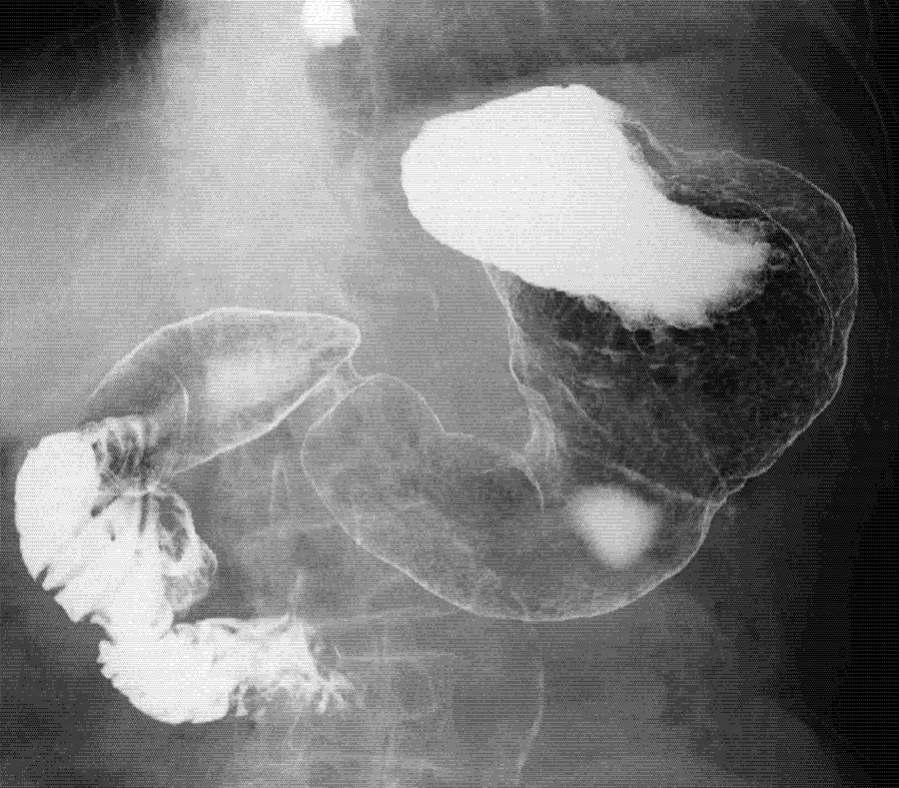
Postoperative upper gastrointestinal fluoroscopy showed little gastric deformity, good peristalsis, and the smooth flow of contrast agent into the duodenum

Her postoperative course was good. She started oral intake on postoperative day 1, and was discharged home on postoperative day 7. Postoperative upper gastrointestinal fluoroscopy showed little gastric deformity, good peristalsis, and the smooth flow of contrast agent into the duodenum (Fig. 4). Six month post-surgery, her bodyweight was the same as before surgery, her hemoglobin was increased and maintained at 12, her serum albumin level was normal, and there was no recurrence on computed tomography. She has survived for 2 years after surgery without obvious recurrence or other illnesses, and with no interference to her daily life or dietary intake.

## Discussion

According to the report, “Future Estimates of Cancer Incidence, Mortality, and Morbidity in Japan”, the incidence of gastric cancer will not change significantly, but the proportion of people aged 75 and older will increase significantly [3]. In these elderly patients, various comorbidities might be contraindications for radical surgery.

The radical gastrectomy for AGC results in reduced gastric volume and food intake, often leading to weight loss and decreased muscle mass. In addition, disorders, such as abdominal bloating, diarrhea, and dumping symptoms, may occur after gastrectomy, significantly reducing quality of life [4, 5]. Moreover, the postoperative complication rate appears to be high in elderly patients after gastrectomy [6, 7]. Thus, for elderly patients, it may be appropriate to reconsider their treatment strategy, with respect to the balance between quality of life and prognosis.

In the case of follow-up without curative surgery, such as best supportive care, it is often necessary to control the symptoms of AGC, such as bleeding and stenosis. For these cases, the gastro-jejunal bypass, radiation therapy and palliative gastrectomy without standard lymphadenectomy are often indicated. However, the risk of bleeding cannot be eliminated completely with gastro-jejunal bypass or palliative radiation therapy. Therefore, we consider LECS to be a good candidate for palliative treatment, as it is likely to suppress the symptoms of the gastric tumor with minimal invasiveness.

In 2014, LECS for submucosal tumors was covered by the Japanese National Health Insurance plan, and in 2020, gastric cancer was also covered. Classical LECS carries the risk of abdominal infection, scattering of tumor cells in the abdominal cavity, and dissemination of tumor cells in the peritoneum. However, the development of non-tumor-exposed LECS procedures, such as inverted-LECS, non-exposed endoscopic wall-inversion surgery (NEWS), and closed-LECS, has almost resolved these drawbacks [8]. Although, there are no reports of disseminated recurrence of gastric cancer after LECS, a careful selection of indications and careful post-operative follow-up should be demanded [9–11].

Here, we shed light on a new indication for LECS—"LECS as a palliative treatment of gastric cancer in the elderly". LECS as a palliative surgery was first reported by Takechi et al. in 2018 for a 68-year-old patient [12]; our patient was 89 years. Recently, Nunobe et al. used the National Gastric Cancer Registry to investigate the prognosis of 68,353 patients after Stage I gastric cancer resection, and found that deaths due to diseases other than gastric cancer were significantly increased in elderly people over 75 years who underwent conventional gastrectomy [13]. Furthermore, in this study, 2.6% of Stage I gastric cancer patients in this age group died due to a disease other than gastric cancer within 90 days after a radical gastrectomy [13]. These results show excessive surgical stress has a negative impact in terms of overall survival of patients who undergo radical gastrectomy for gastric cancer.

“LECS as a palliative treatment of gastric cancer in the elderly” has several issues that still need to be clarified. Based on our experience with LECS so far, we believe that there are a number of technical selection criteria necessary for the safe completion of LECS for gastric cancer. First, the size of the cancer should be 5 cm or less, because if the defect in the stomach wall after tumor resection is too large, there may be a high degree of deformation after reconstruction resulting in a delay in gastric emptying. Second, AGC, such as types 3 and 4, might not be indicated for LECS procedures, because the tumor margins are usually difficult to recognize. Third, the location of the tumor is important, as tumors located near the esophagogastric junction or pyloric ring are technically demanding and should not be treated with LECS. In addition, a wide disconnection of the nerve of Latarjet interferes with gastric peristalsis, so LECS should not be planned if the tumor extends widely to both the anterior and posterior walls of the stomach.

However, most importantly, the patient should not be offered palliative LECS in the first instance. Currently, LECS is an option that should only be offered after the patient has refused standard curative treatment. In this current case, our patient totally refused radical gastrectomy for gastric cancer. Other than best supportive care, the only treatment she agreed to was LECS. As a result of the LECS, she has been able to live her desired life at home for 2 years.

Another technique used in LECS is also important for producing a cancer-negative dissection margin. We perform a full-circumferential negative biopsy before the surgery to determine the minimum required dissection line. Furthermore, the preoperative intra-luminal endoscopic mucosal ablation marked by electrocautery makes the dissecting line more accurate. In contrast, a simple wedge resection which is the most popular procedure for gastric local resection might result in cancer-positive margins because of the difficulty in determining the extent of the lesion from the outside of the stomach wall [14].

Furthermore, there are several modified LECS procedures which do not expose the tumor to the abdominal cavity, such as NEWS, closed-LECS, and CLEAN-NET (combination of laparoscopic and endoscopic approaches to neoplasia with a non-exposure technique). Despite the excellent performance of these procedures, they are not easy to perform for every tumor location and size. That is why we used the gastric content-scatterless procedure named “inverted-LECS” in this case, which sufficiently prevents intraoperative cancer cell seeding. Inverted-LECS is technically easy, and it can be performed regardless of tumor location or size.

Local control of AGC with LECS, in terms of preventing tumor bleeding or stenosis, might be an option for palliative treatment of patients with gastric cancer in the future. To prove the safety and efficacy of palliative LECS for gastric cancer as a less invasive surgery, further large-scale studies are warranted.

## Conclusions

Herein, we have presented our experience with a patient undergoing local gastric resection with LECS as palliative surgery for AGC. This procedure might be an option for patients who refuse radical resection for gastric cancer.

## Data Availability

Not applicable.

## Abbreviations

LECS: Laparoscopy endoscopy cooperative surgery
AGC: Advanced gastric cancer
NEWS: Non-exposed endoscopic wall-inversion surgery
